# Toward a Systematic Assessment of Sex Differences in Cystic Fibrosis

**DOI:** 10.3390/jpm13060924

**Published:** 2023-05-31

**Authors:** Christiane Gärtner, Jörg Fallmann, Peter F. Stadler, Thorsten Kaiser, Sarah J. Berkemer

**Affiliations:** 1 Neuromorphic Information Processing, Institute of Computer Science, Leipzig University, Augustusplatz 10, D-04109 Leipzig, Germany; 2 Bioinformatics Group, Institute of Computer Science, Interdisciplinary Center of Bioinformatics, Leipzig University, Härtelstraße 16-18, D-04107 Leipzig, Germany; 3 Academic Department of Laboratory Medicine, Microbiology and Pathobiochemistry, Medical School and University Medical Center East Westphalia-Lippe, Hospital Lippe, Bielefeld University, Röntgenstraße 18, D-32756 Detmold, Germany; thorsten.kaiser@uni-bielefeld.de; 4 LIX CNRS UMR 7161, Ecole Polytechnique, Institut Polytechnique de Paris, 91120 Palaiseau, France; 5 Earth-Life Science Institute, Tokyo Institute of Technology, 2-12-1-I7E-318 Ookayama, Tokyo 152-8550, Japan

**Keywords:** sex bias, gender gap, SABV, cystic fibrosis, differential gene expression, cAMP signaling pathway, estrogen signaling pathway

## Abstract

(1) Background: Cystic fibrosis (CF) is a disease with well-documented clinical differences between female and male patients. However, this *gender gap* is very poorly studied at the molecular level. (2) Methods: Expression differences in whole blood transcriptomics between female and male CF patients are analyzed in order to determine the pathways related to sex-biased genes and assess their potential influence on sex-specific effects in CF patients. (3) Results: We identify sex-biased genes in female and male CF patients and provide explanations for some sex-specific differences at the molecular level. (4) Conclusion: Genes in key pathways associated with CF are differentially expressed between sexes, and thus may account for the gender gap in morbidity and mortality in CF.

## 1. Introduction

Cystic fibrosis (CF) is the most common inherited disease in the Caucasian population [1]. CF is caused by a mutation(s) in the cystic fibrosis transmembrane conductance regulator (CFTR) protein, which results in defects in the expression or activity of a chloride channel located in the cell membrane. CF affects all exocrine organs but mainly affects the lungs [2]. Severe symptoms include decreased chloride secretion, reduced airway surface liquid height, and increased mucus viscosity. This can lead to a range of CF-related health issues such as bacterial proliferation, sustained inflammation, epithelial lung tissue injury, fibrosis, and remodeling. Further problems include the development of CF-related diabetes (due to destroyed islets of Langerhans), high infertility rates, and malnutrition, as well as low socioeconomic status and decreased quality of life [2].

CF is a disease with well-documented clinical differences between female and male patients. This “gender gap”, however, is very poorly studied at the molecular level. We are aware of only a single study that addresses sex-biased gene expression in CF patients [3]. This contribution summarizes the current knowledge of sex-related differences in CF and analyzes RNA-seq data to identify patterns of differential gene expression that may be related to the known differences in clinical outcomes. Furthermore, it shows that the pathways that play key roles in CF and harbor genes with sex-biased expression can provide explanations for some sex-specific differences.

### 1.1. Sex Differences in CF

Women experience more severe effects of CF. The observed sex-related differences related to morbidity and mortality in CF include an extended length of hospitalization for women due to more complex pulmonary exacerbation treatment regimens. This may be a direct effect of the higher infection rate observed in women for, e.g., *Pseudomonas aeruginosa* or methicillin-resistant *Staphylococcus aureus* (MRSA) [4]. CF-related diabetes (CFRD) can lead to more severe complications, a more significant decline in lung function, and an increased mortality rate in women [5,6]. All the observed effects of CF contribute to a lower median survival age in women (49 years) compared to men (56 years) [7]. These sex-related differences have also been reported in other respiratory diseases [8]. Despite the existence of anatomical differences [9], sex hormones are assumed to be responsible for most of the symptoms, with significantly different effects observed between men and women [2].

### 1.2. Possible Explanations for the “Gender Gap”

Anatomical differences, in particular, the reduced airway diameter and lung volume in women, have been described as putative reasons for the differences in CF symptoms and complications [2,9]. Other differences have been described that can exacerbate asthma and autoimmune conditions in women such as more robust T-cell immunity, exuberant T-helper (Th)-2 response, eosinophilic inflammation, and IL-33 production [2]. Further risk factors include nutritional differences, which result in a body mass index (BMI) that is lower in female CF patients [4]. Social and behavioral studies show that women are more resistant to nutritional interventions and experience poorer medication and nutritional adherence. It is well-known that a reduced BMI and poor nutritional status contribute to decreased lung function and increased mortality [2].

### 1.3. Influence of Sexual Hormones on CF

Despite all the above-mentioned anatomical, social, and behavioral factors, recent developments show that puberty has an effect on CF and strongly indicate that being of the female sex is an independent risk factor in CF patients [10,11]. The following summarizes the recent results of studies regarding the influence of sex hormones on CF cells. For a more detailed summary of existing animal and human studies, see Table 1 in [2].

The effects of estrogen are mediated via estrogen receptors 1 and 2 (ESR1 and ESR2) and the 7-transmembrane G protein-coupled estrogen receptor (GPER). ESR1 and ESR2 are ligand-activated transcription factors that affect, e.g., proteins involved in cell cycle regulation and inflammation. On the other hand, GPER interacts with the epidermal growth factor receptor (EGFR) in transactivation, and it also influences a diverse array of signaling pathways [12]. In a microarray study by Clarke et al. [13] in human native nasal epithelial cells from CF patients compared to non-CF controls, ESR1 was upregulated 1.84-fold in CF patients. Additional targets for ESR1 were significantly enriched in samples from CF patients, whereas differential expression levels of both ESR2 and GPER were not observed.

In vitro tests have shown that estrogen decreases the innate immune response to bacterial infections [2]. In mice, the increased expression of IL-23 and IL-17 has been reported in response to estradiol stimulation in CFTR-deficient cells [14]. Hence, estrogen may directly alter bacterial behavior. In an in vitro study, it was shown that estrogen promotes the growth of *P. aeruginosa*, and this effect may vary depending on the phase of the menstrual cycle. Moreover, estrogen was found to reduce the intensity of ciliary beat frequency [15]. In another study, serum estradiol levels were found to be significantly higher during periods of CF exacerbation [16]. Further in vitro studies in human CF bronchial epithelial cells identified an estradiol-dependent upregulation of SLPI and an inhibition of IL-8, together with decreased ciliary beat frequency and reduced chloride transport in a CFTR-independent manner.

In a study focusing on the clinical outcomes for humans (no gene expression analyses), it was shown that treatment with Ivacaftor (CFTR potentiators) resulted in reduced rates of pulmonary exacerbation (PEx) in females. However, no significant sex-related differences were observed in terms of changes in forced expiratory volume in one second (FEV1) or body mass index (BMI) after the conclusion of the study [17].

## 2. Materials and Methods

### 2.1. Data Source

Data used in this study were obtained from the NCBI GEO database [18] under accession number GSE205161 [19]. The available data have been filtered to include only individuals without nontuberculous mycobacterial pulmonary disease infections. Additionally, individuals aged 18 and above were chosen to guarantee full adolescence. Next, the datasets were filtered to include individuals of Caucasian heritage, as this was a predominant factor in the available datasets and allowed for better comparison of medical parameters regarding lung status. Of the remainder, 5 female and 5 male whole blood transcriptomics bulk RNA-seq datasets were chosen randomly for this study. The available baseline characteristics of the patients subdivided according to sex are listed in Table 1.

The numbers of uniquely mapped reads were similar between sexes, with the exception of an upwards outlier in female sample no. SRR19451822, which contained 2–4× we confirm the change. more unique mappers compared to the other samples. However, in terms of the relative numbers, all samples contained between 60% and 75% unique mappers.

### 2.2. Data Processing and Analysis

The downloading, processing, and analysis of the data used in this study were conducted using MONSDA [20] release v1.1.0. The corresponding configuration file is available at https://raw.githubusercontent.com/jfallmann/MONSDA_companion/main/CysticFibrosisGenderBias/config_cystic_fibrosis_monsda.json, accessed on 26 May 2023.

#### 2.2.1. Pre-Processing and Mapping

GEO Series GSE205161 was downloaded using sra-tools [21] version 2.11.0. The raw reads were trimmed using cutadapt [22] version 4.1 and mapped using star [23] version 2.7.10b. Quality control was conducted for the raw, trimmed, and mapped reads using FastQC [24] version 0.11.9 and MultiQC [25] version 1.14.

#### 2.2.2. Detection of Differentially Expressed Genes between Females and Males

To identify differentially expressed genes, the uniquely mapped reads were quantified using FeatureCounts [26] version 1.6.4 and analyzed for DE using DESeq2 [27] version 1.32.0. The expression of genes from GENCODE v37 was compared between CF-diseased female and male samples, with the former regarded as the baseline condition. Genes that were significantly (absolute fold-change > 2, FDR < 0.05) differentially expressed were classified into two categories, male-biased genes (MG) and female-biased genes (FG), based on their overexpression in comparison to the other condition [28]. Therefore, MG is the set of genes overexpressed in CF-diseased males compared to CF-diseased females, and vice versa for FG.

#### 2.2.3. Detection of Sex-Biased Alternatively Spliced Genes

Alternative splicing analysis was conducted using EdgeR [29] version 3.28.0, following the quantification of the exon expression using FeatureCounts [26] version 1.6.4. The definitions of FG and MG are consistent with the information presented in Section 2.2.2.

#### 2.2.4. Network analysis and subcellular locations

The downstream identification and analysis of pathways affected by differentially expressed genes were conducted using StringDB [30] and KEGG mapper [31,32]. All the steps in the analysis were conducted with the default settings and cutoffs.

## 3. Results

### 3.1. Differentially Expressed Genes in Female versus Male Patients with Cystic Fibrosis

**Figure 1:**
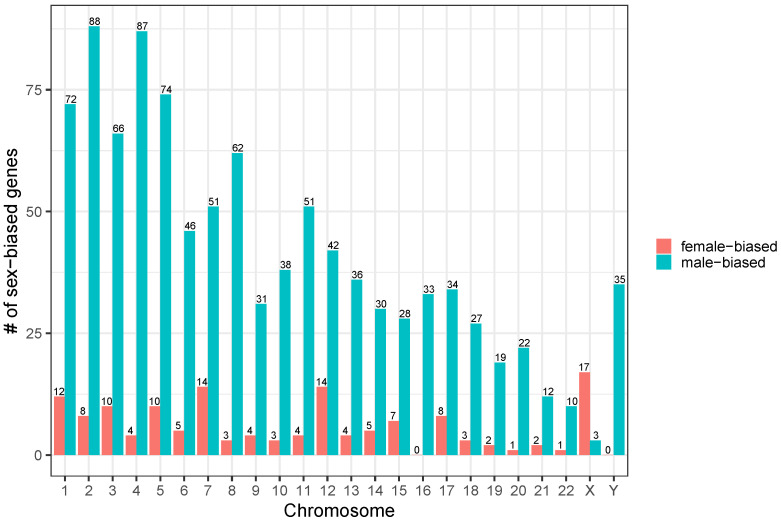
Chromosomal distribution of male-biased and female-biased upregulated genes in cystic fibrosis patients.

We found 1140 genes expressed in a sex-biased manner: 999 male-biased genes (MG) and 141 female-biased genes (FG). The distribution of FG and MG across the different chromosomes is shown in Figure 1. Overall, most sex-biased genes were MG and were found in autosomes; however, some MG were found on the X chromosome. The relative distribution of sex-biased genes to the total number of genes of a chromosome showed that most sex-biased genes were located on chromosome 4 and chromosome 13.

### 3.2. Enrichment Analysis of Sex-Biased Expressed Genes in CF

During the investigation of enriched interactions, pathways, cellular processes, and cellular compartments within the set of sex-biased expressed genes, significant enrichments of pathways and cellular functions were observed, which provide some insights into CF-specific sex biases. The results are summarized in Table 2.

#### 3.2.1. Pathways

Ideozu et al. [33] summarized several expression studies in CF and showed the following key pathways in CF: cytokine signaling, inflammatory response, cell-to-cell signaling, TLR signaling, chemokine signaling, AMPK-Akt signaling, glycosylation of biopolymers, E1F2 signaling, and IL-8 signaling. Regarding sex-biased genes in CF, our results align with theirs in terms of glycosylation, cell-to-cell signaling, multicellular organismal signaling, cAMP signaling, and the estrogen pathway, as shown in Table 2.

The expression differences of genes encoding for estrogen receptors 1 and 2 (ESR1 and ESR2) were not significant between the sexes according to the strict logFC cutoffs. However, the general expression of ESR2 was very high (base mean 1576) and the logFC at −0.79 was significant without the cutoffs (adjusted *p*-value < 0.001). Therefore, assuming relevant effects is not unfounded. ESR1 was also expressed at a high level, although the level was lower compared to ESR2 (base mean 540) with a logFC of −0.33. In this case, no statistical significance (adjusted *p*-value 0.12) was found. G protein-coupled estrogen receptor (GPER1) was found to be expressed only at low levels (base mean 25.1), without any fold changes observed between the sexes.

In addition, genes involved in inflammatory processes (IL-17 signaling) were found to exhibit sex-biased expression. Among the FG, we detected CXCL5, a known chemokine with IL-17 upregulating function. Among the MG, genes were identified that affect the O-glycosylation of TSR domain-containing proteins. These genes play a role in regulating inflammation and are involved in the functioning of NOD-like receptors, which are essential for recognizing pathogen-associated molecular patterns that are crucial for infection response.

#### 3.2.2. Subcellular Location of Sex-Biased Genes

**Figure 2:**
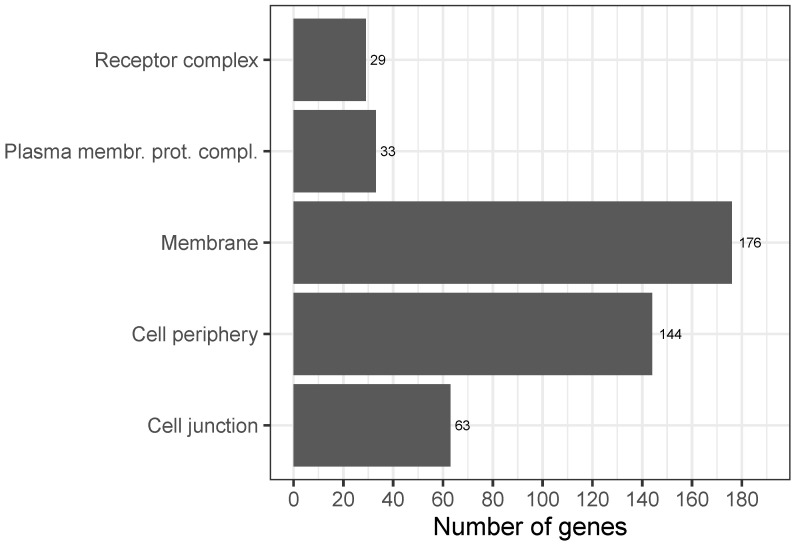
Sex-biased expressed genes in cystic fibrosis and their distribution across cellular compartments (selection, multiple assignments per gene possible). For detailed gene lists, see Table A3 and Table A4.

Enrichment analysis showed that the sex-biased genes were located close to or inside the cell membrane, with some genes even being integral components of the membrane. In addition, most of them were MG (18 FG vs. 111 MG), as depicted in Figure 2. Enrichment of this cellular compartment appears conclusive since CF is based on a mutation of an ion channel localized in the cell membrane.

### 3.3. Alternatively Spliced Genes between Female and Male Patients with Cystic Fibrosis

Table A5 and Table A6 list the genes that were detected as differentially expressed in male and female CF patients. All of these genes showed enrichment in pathways related to alternative splicing based on the results from StringDB. In contrast, our own analysis only showed 33 alternatively spliced genes regarding sex bias (16 MG, 17 FG; see also Table A7 and Table A8). No overlaps between the StringDB results and our set of genes were detected. This is not surprising because the comparison was performed on two levels. The list of MG and FG differentially expressed genes contained some genes that regulate the alternative splicing process, whereas this analysis detected genes that are potential targets of alternative splicing.

## 4. Discussion

Cystic fibrosis is a disease with well-documented clinical differences between female and male patients. In this contribution, we analyzed the RNA-seq data of five female and five male CF patients in order to detect differentially expressed genes regarding patient sex. The results demonstrate the presence of significantly sex-biased genes. Therefore, sex-specific clinical treatment and medication may decrease differences in clinical outcomes and symptoms and improve the living conditions of CF patients regardless of gender.

There was minimal overlap observed when comparing the overall differential gene expression between healthy female and male subjects, similar to what was found in a study by Talebizadeh et al. [34] on X chromosome gene expression in female and male tissues. Comparing sex-biased genes in healthy individuals revealed a minimal overlap with those identified in CF-affected individuals. Most sex-biased expressed genes in CF patients were not found to be differentially expressed between healthy individuals of both sexes, which highlights the need for specific studies, as the transferability of results is not guaranteed. Only 30 out of 883 FG and 21 out of 894 MG were found in peripheral mononuclear blood cells, overlapping with those of healthy subjects described in Guo et al. [28]. Therefore, a CF-specific sex bias can be derived.

Ogilvie et al. reported a total of 863 differentially expressed genes between bronchial epithelium samples of CF vs. healthy individuals [35], only a fraction of which were found in this study. This was also the case when comparing our results to genes differentially expressed between CF and non-CF patients in the microarray study by Clarke et al. [13], where only a few additional genes were sex-biased expressed. Acknowledging the differences between the technologies used in these studies and the analysis presented here (microarray vs. RNA-Seq), as well as the difference in the tissue under consideration (epithelium vs. plasma), we conclude that sex plays an important role as a confounding factor in differential expression analysis and should not be ignored. Furthermore, the differences between sexes seem to be even more pronounced than the overall differences observed between pooled healthy and diseased subjects.

In severe CF, differentially expressed genes regarding CF vs. non-CF patients were reported to be involved in protein ubiquitination, mitochondrial oxidoreductase activity, and lipid metabolism [36]. Our study identified an overlap in sex-biased expressed genes, specifically CALR3 (Calreticulin, Ca2 binding, and storage) as a male-biased expressed gene and IGFBP3 as a female-biased expressed gene.

When comparing our results with other studies on the gender gap in CF, we found no overlap between the miRNAs involved in inflammatory processes [3] and the sex-biased miRNAs detected here or the list of hub genes identified in the meta-analysis by Trivedi et al. [37]. In that study, a hub gene analysis was conducted on microarray-derived gene expression datasets and hub genes were identified based on a protein–protein interaction network. As stated by the authors, the identified hub genes (MYC, EZR, S100A9, S100A8, TF, TIA1, KYNU, KLF6, CSTA, and LRRFIP1) were present in the IL-17 signaling pathway and the mineral absorption and gastric-acid secretion pathways [37]. However, the study focused on healthy vs. affected individuals and we found no overlaps with the sex-biased genes identified in our study. This implies that hub gene expression is not sex-biased within CF patients. Given their central role in sex-independent molecular mechanisms, this is not surprising.

Analysis of the subcellular location of sex-biased genes, which were mostly MG, showed a strong trend toward localization in or around the cell membrane. We hypothesize that an increased expression of different ion channels in males, especially CFTR, leads to a better compensatory response for the mutated CFTR protein (see Table A3 and Table A4).

### 4.1. IL-17 Signaling Pathway

In their study using Luminex multiplex assays, Deny et al. [3] reported that a few inflammatory mediators were more highly expressed in the plasma of females (TNF-α, IL-1b, IL-8, IL-10, IL-12p70, IL-17A, and CXCL10). They mainly appeared in the IL-17 pathway (TNF-α, IL-1b, IL-17A, and CXCL10) involved in the response to inflammation and as a host defense. As described above, our results also included genes involved in the IL-17 pathway (MAPK and MMP9 (both MG) and CXCL5 (FG)). Here, MMP9 is responsible for tissue remodeling, hence upregulation in male patients may cause severe effects. Vermeer et al. [38] reported that higher expression of MMP9 in asthma patients was shown to decrease the functionality of cell barriers (specifically the tight junction pathway), which can limit the protection against bacteria entering the cells. However, MMP9 was shown to correlate adversely with lung function in CF patients [39] and has been discussed as a potential future therapeutic target in CF [40]. Female-biased expression of the chemokine CXCL5, also part of the IL-17 signaling pathway, could partly account for more intense inflammation reactions in females.

The dataset used in this study consists of whole blood transcriptomes, whereas cystic fibrosis is known to affect mostly epithelial cells. However, the IL-17 signaling pathway, as well as the pathways described in the following subsections, play significant roles in signaling cascades in both epithelial and whole blood cells. IL-17 can be produced by a wide range of immune cells present in the blood, e.g., Th17 cells, natural killer T cells, group 3 innate lymphoid cells, CD8+ cells, and neutrophils; [41], whereas IL-17 receptor A is widely expressed in epithelial cells, fibroblasts, and blood cells, e.g., macrophages, dendritic cells, and peripheral blood T lymphocytes [42]. The IL-17 downstream pathways induce the production of inflammatory molecules and chemokines, which leads to the recruitment of inflammatory cells. This process mediates the inflammatory response and contributes to the genesis of autoimmune disorders [41].

### 4.2. cAMP Signaling Pathway

**Figure A1:**
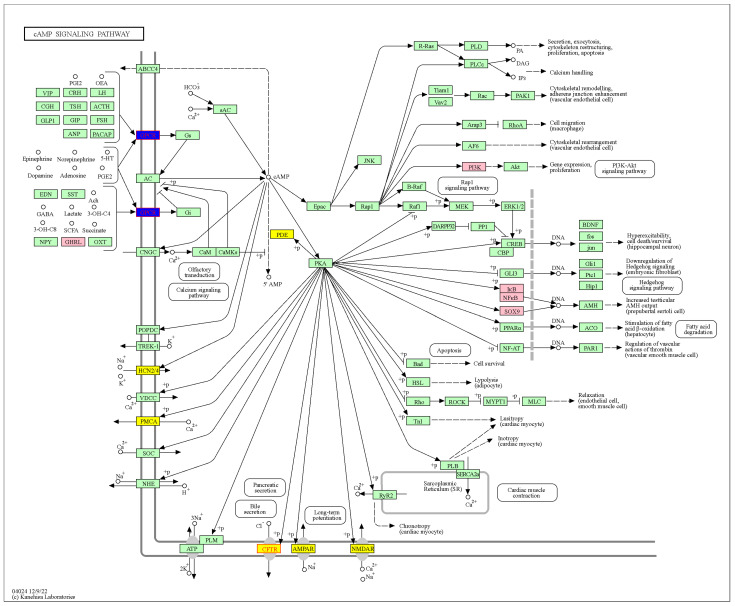
Sex-biased expressed genes in cystic fibrosis and their involvement in the cAMP signaling pathway (from KEGG Mapper). **Pink** indicates differentially expressed genes in CF vs. non-CF [3], **yellow** indicates sex-related differentially expressed genes in CF patients detected here (all MG, see also Table 2), and **blue** indicates potential targets for therapeutics (β-2 adrenergic receptor agonists (bronchodilatation): ADRB2), as described in the main text. The CFTR gene (**yellow/red**) is the gene whose mutation causes CF. It appears as a male-biased gene but is also used as a target in therapeutics to restore activity. The double line represents the cell membrane. Genes marked in yellow: HCN4, GRIN2A, GRIN2B, GRIA1, GRIA2, ATP2B2, PDE10A. Genes marked in blue: ADRB2. Genes marked in pink: GHRL, PIK3R1, NFKBIA, NFKB1, SOX9. Genes marked in yellow/red: CFTR.

The cAMP signaling pathway regulates homeostasis through direct or indirect modulation of transmembrane ion channels, including those for Ca^2+^, Na^+^, and K^+^, as well as CFTR via PKA activation. In our dataset, CFTR is a male-biased gene, suggesting a potential mechanism in males to compensate for the loss of function due to mutations. The beta-adrenoreceptor agonists are known as therapeutic targets in CF and act on the cAMP pathway via ADRB2 [43]. Figure A1 depicts the cAMP pathway (KEGG mapper). The genes mentioned in our study are marked in different colors. The targeted ADRB2 gene is marked in blue, yellow indicates the sex-biased genes detected in our study (all MG; see also Table 2), and pink indicates differentially expressed genes between CF and non-CF patients reported by other studies. The CFTR gene is marked in yellow/red and is located in the lower part of the figure (inside the cell membrane).

Adenylate cyclase (AC) produces cAMP, which affects ciliary beat frequency (lower in CF). AC function is impaired in CF but is more highly expressed in males, which may partly compensate for the lower functionality in CF [44]. The importance of the cAMP signaling pathway in CF is evident, as the inhibition of cAMP degradation via different phosphodiesterase inhibitors has been discussed and investigated as a therapeutic target (summarized in [45]). Sun et al. [46] reported that SNPs in ATP2B2 showed an association with the development of meconium ileus in CF patients.

Notably, all sex-biased expressed genes involved in the cAMP signaling pathway were found to be MG and mostly encode proteins located within or at the cell membrane, directly influencing cell homeostasis via ion channel regulation. Some of them are part of tight junctions or play a role in their regulation, with the latter being important in maintaining the function of epithelial tissue of the lungs, among others. However, we also observed a direct impact of cAMP signaling on whole blood cells. Chronic inflammation is characterized by the excessive migration of leukocytes from the peripheral blood into the tissues. The leukocyte extravasation process is regulated by signaling pathways in both leukocytes and vascular endothelium, which involve cAMP and calcium as intracellular messengers [47].

### 4.3. Estrogen Pathway

**Figure A2:**
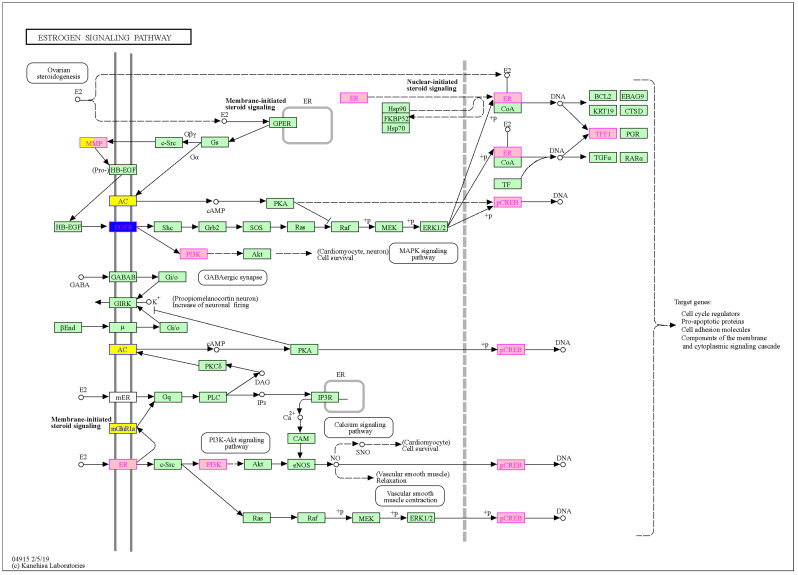
Sex-biased expressed genes in cystic fibrosis and their involvement in the estrogen signaling pathway (from KEGG Mapper [31]). Estrogen acts either via estrogen receptors 1 and 2 (mER, ER) or via the G protein-coupled estrogen receptor (GPER). **Yellow** indicates differentially expressed genes regarding CF patient sex, which are all MG (see also Table 2). **Pink** indicates differentially expressed genes in CF vs. non-CF conditions [3]. **Blue** indicates EGFR, which is a potential target for therapeutics and is also regarded as a male-biased gene in our data (see also Table 2 and the main text for more details). The gene marked in **yellow/pink** (MMP9) is a male-biased gene, more highly expressed in CF vs. non-CF, and occurs in the IL-17 signaling pathway. Genes marked in pink: ESR1, TFF1, PIK3R1, ATF2, ATF4, ATF6B. Genes marked in yellow/pink: MMP9. Genes marked in blue: EGFR. Genes marked in yellow: ADCY2, ADCY8, GRM1, (EGFR).

When comparing the sex-biased genes identified in this study to genes involved in the estrogen pathway, an overlap was observed only for MG (see also Figure A2). However, genes can be considered *protective* if expressed at higher levels. As reported by Kim et al. [48], non-mutated CFTR suppresses airway epithelial IL-8 production that occurs via a stimulatory EGFR cascade. A loss of normal CFTR activity exaggerates IL-8 production via the activation of a pro-inflammatory EGFR cascade. Both CFTR and EGFR are MG, possibly resulting in lower IL-8 production and less aggressive inflammation in males. This indicates a plausible protective characteristic and further hints at the stimulation of ciliary beat frequency, which is otherwise impaired by low adenylate cyclase levels.

Changes in the expression of genes involved in the estrogen pathway can also directly influence blood cells. Neutrophils, monocytes, macrophages, and lymphocytes, among others, express estrogen receptors [49], suggesting that estrogen directly affects the function of these cells. Estrogen has been found to suppress the development of B cells, but on the other hand, it has also been found to augment B cell functions, eventually leading to higher levels of antibodies observed in females. The number of circulating neutrophils is also influenced by the different levels of estrogen present during the different phases of the menstruation cycle in females [50]. Interestingly, estrogen treatment was shown to increase Th17 cells in the early phase of collagen-induced arthritis [51]. As IL-17 is essential for Th17 cells, this indicates a tight connection between estrogen and the IL-17 pathways.

### 4.4. Calcium Signaling Pathway

As shown in Table 2, a large number of MG genes were found to be involved in the calcium signaling pathway, many of which were located within the membrane of the cell. Here, EGFR, which is also involved in the estrogen pathway, and ADRB2, which is also involved in the cAMP pathway, were found to play a role in the calcium signaling pathway, indicating their involvement in multiple pathways. Moreover, calcium signals are also crucial for blood cells, such as those investigated in this study. They mediate the production of cytokines and the reprogramming of T cells, leading to their differentiation into various T cell subsets [52].

### 4.5. Further Targets

A known and reported target for future CF therapeutics [53,54] is the gene ANO1, which encodes for TMEM16A, an anion-selective channel activated by the binding of Ca^2+^ from the cytoplasm. ANO1 (logFC 1.9) was identified as MG in our data; therefore, targeting ANO1 may lead to different outcomes for male and female patients.

### 4.6. Outlook

Although Basu et al. [55] reported that approximately 75% of differentially expressed genes from whole blood analysis can be transferred to lung tissue, the field would benefit from a dedicated follow-up study that analyses sex-biased genes in bronchial epithelium. Halloran et al. showed a strong correlation (Spearman’s rank correlation coefficient of 0.9) between mean expression levels in lung tissue and whole blood samples [56]. According to a study by Rotunno et al., the gene expression signature derived from blood samples reflects cancer-related gene expression changes in lung tissue, particularly in stage I lung adenocarcinoma [57]. A similar study was conducted to differentiate groups of patients with idiopathic pulmonary fibrosis, where a 13-gene cluster expressed in blood was used as a classifier to separate the groups. Yang et al. [58] presented a similar method to distinguish individuals with idiopathic pulmonary fibrosis from others.

Our analysis revealed that alternative splicing was a mechanism regulated by many of the sex-biased differentially expressed genes. Although our investigation only detected a small number of these sex-biased alternatively spliced genes, we conclude that further investigation into this topic is worthwhile. Datasets with higher sequencing depth would provide more statistical power and should be combined with dedicated analysis workflows to analyze this mechanism and its role in the regulation of sex-biased gene expression in more detail.

## 5. Conclusions

Our results show that some important pathways in CF pathology are MG expressed (IL-17 signaling pathway, cAMP signaling pathway, estrogen pathway, and calcium signaling pathway) and thus can account for the gender gap in mortality and morbidity in CF. The low number of sex-specific patient samples that limits our results emphasizes the importance of sex-specific studies in CF and other diseases to develop therapeutics that take into account patients’ sex and the corresponding biases.

## Figures and Tables

**Table 1:** Baseline characteristics of males and females in the dataset used. Results are presented as medians (interquartile range). There were no significant differences observed in the baseline characteristics using Wilcoxon’s rank sum test (p>0.05). FEV_1_: forced expiratory volume in one second; FVC: forced vital capacity.

	Females	Males
Patients (n)	5	5
Age (years)	32 (30; 38)	29 (26; 33)
FEV_1_ (% predicted)	89.1 (80.6; 94.6)	63.7 (45.6; 83.0)
FEV_1_/FVC	0.79 (0.78; 0.8)	0.65 (0.56; 0.76)

**Table 2:** Genes and corresponding KEGG pathways exhibiting sex-biased expression in CF.

Pathway/Function	MG	FG
hsa04657 IL-17 signaling pathway	MAPK4, MMP9	CXCL5
hsa04915 Estrogen signaling pathway	MMP9, ADCY2, ADCY8, EGFR, GRM1	–
hsa04152 AMPK signaling pathway	PPP2R2C, CFTR	–
hsa04024 cAMP signaling pathway	FSHR, ADCY2, ADCY8, HCN4, GRIN2A, GRIN2B, GRIA1, GRIA2, CFTR, ATP2B2, PDE10A	–
hsa04020 Calcium signaling pathway	ATP2B2, ADCY2, ADCY8, GRIN2A, TRDN, CASQ2, ADRA1B, GRM1, GRM5, FGF1, NGF, EGFR, ERBB4, NTRK2, PDE1A, PDE1C	MCOLN2 (TRPML)
hsa04530 Tight junction	CLDN14, MPP4, PPP2R2C, CGNL1, CFTR, EPB41L4B, MAGI1	CLDN20, DLG3
hsa5173214 O-glycosylation of TSR domain-containing proteins	ADAMTS18/19/20, ADAMTS3, ADAMTSL1, ADAMTS7, ADAMTS12	–

## Data Availability

Not applicable.

## Abbreviations

The following abbreviations are used in this manuscript:

CF	cystic fibrosis
FG	female-biased genes
MG	male-biased genes
hsa	homo sapiens
CFTR	cystic fibrosis transmembrane conductance regulator
SABV	sex as biological variable
ESR	estrogen receptor
GPER	G protein-coupled estrogen receptor

## Appendix C. Overlap with StringDB

**Table A1:** Male-biased genes (MG) in CF found in StringDB associated with various cellular functions.

Gene Name	Term Description	Term ID	Gene Name	Term Description	Term ID
NALCN	Voltage-gated ion channel activity	GO:0005244	ANO3	Transporter activity	GO:0005215
ABCC9	Signaling receptor activity	GO:0038023	GABRA4	Signaling receptor activity	GO:0038023
GABRA6	Signaling receptor activity	GO:0038023	KCNE4	Voltage-gated ion channel activity	GO:0005244
KCNT2	Voltage-gated ion channel activity	GO:0005244	TMC1	Voltage-gated ion channel activity	GO:0005244
CACNG2	Voltage-gated ion channel activity	GO:0005244	KCNH5	Voltage-gated ion channel activity	GO:0005244
SLC24A2	Calcium channel activity	GO:0005262	SCNN1B	Ligand-gated ion channel activity	GO:0015276
CACNA2D1	Voltage-gated ion channel activity	GO:0005244	TRPC4	Calcium channel activity	GO:0005262
KCNU1	Voltage-gated ion channel activity	GO:0005244	SCN10A	Voltage-gated ion channel activity	GO:0005244
PIEZO2	Transporter activity	GO:0005215	TRPC7	Calcium channel activity	GO:0005262
CACNG5	Voltage-gated ion channel activity	GO:0005244	TMEM120A	Transporter activity	GO:0005215
GABRG3	Signaling receptor activity	GO:0038023	SLC10A2	Transporter activity	GO:0005215
SLC6A15	Transporter activity	GO:0005215	SLC28A1	Transporter activity	GO:0005215
TUSC3	Transporter activity	GO:0005215	SLC6A5	Transporter activity	GO:0005215
SLCO6A1	Transporter activity	GO:0005215	GRM5	Neurotransmitter receptor activity	GO:0099583
GRM1	Neurotransmitter receptor activity	GO:0099583	RANBP17	Transporter activity	GO:0005215
UNC13C	Calcium ion binding	GO:0005509	GUCA1C	Calcium ion binding	GO:0005509
CASQ2	Calcium ion binding	GO:0005509	CDH19	Calcium ion binding	GO:0005509
LRP2	Calcium ion binding	GO:0005509	CDH10	Calcium ion binding	GO:0005509
FSTL5	Calcium ion binding	GO:0005509	SLIT3	Calcium ion binding	GO:0005509
EML1	Calcium ion binding	GO:0005509	PCLO	Calcium ion binding	GO:0005509
DNER	Signaling receptor activity	GO:0038023	SMOC2	Calcium ion binding	GO:0005509
EGFLAM	Calcium ion binding	GO:0005509	SPOCK3	Calcium ion binding	GO:0005509
PCDH15	Calcium ion binding	GO:0005509	TGM3	Calcium ion binding	GO:0005509
LRP1B	Calcium ion binding	GO:0005509	CALN1	Calcium ion binding	GO:0005509
SVEP1	Calcium ion binding	GO:0005509	DGKB	Calcium ion binding	GO:0005509
CLSTN2	Calcium ion binding	GO:0005509	CCBE1	Calcium ion binding	GO:0005509
SLIT2	Calcium ion binding	GO:0005509	CDH18	Calcium ion binding	GO:0005509
TENM2	Calcium ion binding	GO:0005509	FAT3	Calcium ion binding	GO:0005509
PCDH7	Calcium ion binding	GO:0005509	CDH8	Calcium ion binding	GO:0005509
PLA2G4C	Calcium ion binding	GO:0005509	HMCN2	Calcium ion binding	GO:0005509
NOD2	Signaling receptor activity	GO:0038023	PKHD1	Signaling receptor activity	GO:0038023
PAQR5	Signaling receptor activity	GO:0038023	HNF4G	Signaling receptor activity	GO:0038023
ADAMTS19	Metalloendopeptidase activity	GO:0004222	ADAMTS18	Metalloendopeptidase activity	GO:0004222
MMP16	Metalloendopeptidase activity	GO:0004222	ADAMTS3	Metalloendopeptidase activity	GO:0004222
MMP9	Metalloendopeptidase activity	GO:0004222	ADAM32	Metalloendopeptidase activity	GO:0004222
ADAMTS7	Metalloendopeptidase activity	GO:0004222	ADAMTS20	Metalloendopeptidase activity	GO:0004222
ADAMTS12	Metalloendopeptidase activity	GO:0004222	SHISA6	PDZ domain binding	GO:0030165

**Table A2:** Female-biased genes (FG) in CF found in StringDB associated with various cellular functions.

Gene Name	Term Description	Term ID	Gene Name	Term Description	Term ID
MCOLN2	Ligand-gated ion channel activity	GO:0015276	CD69	Signaling receptor activity	GO:0038023
DSC1	Calcium ion binding	GO:0005509	TNNT3	Calcium ion binding	GO:0005509
KRT1	Signaling receptor activity	GO:0038023	DLG3	PDZ domain binding	GO:0030165

**Table A3:** Male-biased genes (MG) in CF found in StringDB associated with different cellular compartments.

Gene Name	Term Description	Term ID	Gene Name	Term Description	Term ID
NTRK2	Intrinsic component of membrane	GOCC:0031224	LHFPL4	Membrane	GOCC:0016020
GRIA2	Intrinsic component of membrane	GOCC:0031224	PCLO	Cytoskeleton of presynaptic active zone	GOCC:0048788
ERBB4	Plasma membrane protein complex	GOCC:0098797	NGF	Somatodendritic compartment	GOCC:0036477
RAB3B	Intrinsic component of membrane	GOCC:0031224	PCDH15	Plasma membrane-bounded cell projection	GOCC:0120025
SYNDIG1	Membrane	GOCC:0016020	ELAVL2	Presynapse	GOCC:0098793
NLGN1	Glutamatergic synapse	GOCC:0098978	SNAP91	Presynaptic membrane	GOCC:0042734
APBB2	Membrane	GOCC:0016020	GRIA1	Glutamatergic synapse	GOCC:0098978
SLC6A5	Membrane	GOCC:0016020	PPFIA2	Dendritic spine	GOCC:0043197
SHANK2	Dendritic spine	GOCC:0043197	CNTNAP4	Presynaptic membrane	GOCC:0042734
GRHL2	Cell junction	GOCC:0030054	EGFR	Intrinsic component of membrane	GOCC:0031224
DLC1	Membrane	GOCC:0016020	SHROOM3	Cell junction	GOCC:0030054
NRAP	Cell junction	GOCC:0030054	FRMPD2	Membrane	GOCC:0016020
MPP4	Cell junction	GOCC:0030054	CTNNA3	Cell junction	GOCC:0030054
CFTR	Intrinsic component of membrane	GOCC:0031224	GPRC5A	Membrane	GOCC:0016020
GNGT1	Plasma membrane protein complex	GOCC:0098797	NALCN	Membrane	GOCC:0016020
FOLH1	Intrinsic component of membrane	GOCC:0031224	MEOX2	Cell periphery	GOCC:0071944
CDH19	Membrane	GOCC:0016020	LRP2	Membrane	GOCC:0016020
SLC6A15	Intrinsic component of membrane	GOCC:0031224	MMP16	Intrinsic component of membrane	GOCC:0031224
KCNT2	Membrane	GOCC:0016020	CSMD3	Membrane	GOCC:0016020
USH2A	Membrane	GOCC:0016020	ADRA1B	Membrane	GOCC:0016020
SLCO2A1	Membrane	GOCC:0016020	OR4N2	Membrane	GOCC:0016020
KCNH5	Membrane	GOCC:0016020	KRT7	Cell periphery	GOCC:0071944
EPHA3	Intrinsic component of membrane	GOCC:0031224	GFRAL	Membrane	GOCC:0016020
SLC24A2	Membrane	GOCC:0016020	SCNN1B	Intrinsic component of membrane	GOCC:0031224
SMOC2	Cell periphery	GOCC:0071944	ANO1	Intrinsic component of membrane	GOCC:0031224
GFRA1	Membrane	GOCC:0016020	TAAR2	Membrane	GOCC:0016020
SLC16A12	Intrinsic component of membrane	GOCC:0031224	NRG3	Intrinsic component of membrane	GOCC:0031224
PTPRT	Intrinsic component of membrane	GOCC:0031224	ITGBL1	Membrane	GOCC:0016020
GPR158	Membrane	GOCC:0016020	TRPM3	Membrane	GOCC:0016020
TGM3	Membrane	GOCC:0016020	NLRP5	Cell periphery	GOCC:0071944
DPP10	Membrane	GOCC:0016020	PTPRZ1	Intrinsic component of membrane	GOCC:0031224
COL25A1	Intrinsic component of membrane	GOCC:0031224	MDGA2	Membrane	GOCC:0016020
KCNU1	Membrane	GOCC:0016020	LRRC32	Intrinsic component of membrane	GOCC:0031224
UNC80	Membrane	GOCC:0016020	BMPR1B	Intrinsic component of membrane	GOCC:0031224
RXFP1	Membrane	GOCC:0016020	LPAR4	Membrane	GOCC:0016020
PEX5L	Intrinsic component of membrane	GOCC:0031224	SLCO6A1	Membrane	GOCC:0016020
TUSC3	Intrinsic component of membrane	GOCC:0031224	CDH18	Membrane	GOCC:0016020
TRPC7	Membrane	GOCC:0016020	SNTG1	Plasma membrane protein complex	GOCC:0098797
PCDH7	Membrane	GOCC:0016020	GPR17	Membrane	GOCC:0016020
CNTN1	Membrane	GOCC:0016020	NRXN3	Intrinsic component of membrane	GOCC:0031224
ABCA8	Membrane	GOCC:0016020	HCRTR2	Intrinsic component of membrane	GOCC:0031224
FGF1	Cell periphery	GOCC:0071944	MLPH	Somatodendritic compartment	GOCC:0036477
TMC1	Plasma membrane-bounded cell projection	GOCC:0120025	OLFM1	Somatodendritic compartment	GOCC:0036477
GRXCR2	Plasma membrane-bounded cell projection	GOCC:0120025	MYO3B	Plasma membrane-bounded cell projection	GOCC:0120025
CASQ2	Intrinsic component of membrane	GOCC:0031224	ARNT2	Receptor complex	GOCC:0043235
LRP1B	Receptor complex	GOCC:0043235	ZPBP	Membrane	GOCC:0016020
ART1	Membrane	GOCC:0016020	SORCS1	Membrane	GOCC:0016020
TYR	Membrane	GOCC:0016020	ABCA12	Intrinsic component of membrane	GOCC:0031224
STXBP5L	Membrane	GOCC:0016020	LRRC4C	Intrinsic component of membrane	GOCC:0031224
FUT9	Membrane	GOCC:0016020	FUT7	Membrane	GOCC:0016020
IGDCC3	Membrane	GOCC:0016020	ROS1	Membrane	GOCC:0016020
GPC5	Intrinsic component of membrane	GOCC:0031224	SEL1L2	Membrane	GOCC:0016020
CADPS	Membrane	GOCC:0016020	TSPAN8	Membrane	GOCC:0016020
CALN1	Membrane	GOCC:0016020	SLIT2	Membrane	GOCC:0016020
REEP1	Membrane	GOCC:0016020	PIK3C2G	Membrane	GOCC:0016020
PLA2G4C	Membrane	GOCC:0016020			

**Table A4:** Female-biased genes (FG) in CF found in StringDB associated with different cellular compartments.

Gene Name	Term Description	Term ID	Gene Name	Term Description	Term ID
CD69	Intrinsic component of membrane	GOCC:0031224	TAS2R5	Membrane	GOCC:0016020
KRT1	Membrane	GOCC:0016020	GPR174	Membrane	GOCC:0016020
TRAT1	Plasma membrane protein complex	GOCC:0098797	ACVR2B	Intrinsic component of membrane	GOCC:0031224
NCR3LG1	Membrane	GOCC:0016020	FPR3	Membrane	GOCC:0016020
MCOLN2	Membrane	GOCC:0016020	GPR183	Membrane	GOCC:0016020
CCDC136	Membrane	GOCC:0016020	KLRC4	Membrane	GOCC:0016020
ABCD2	Membrane	GOCC:0016020	ELOVL2	Membrane	GOCC:0016020
SPTSSB	Membrane	GOCC:0016020	TXLNG	Membrane	GOCC:0016020
KLRC3	Membrane	GOCC:0016020	SLFN12L	Membrane	GOCC:0016020

**Table A5:** Male-biased genes (MG) in CF found in StringDB associated with alternative splicing (StringDB term KW-0025).

Gene Name	Gene Name
GRHL2	DOK5
TYR	RAPGEFL1
DLC1	ARNT2
RBFOX1	SH3GL3
MYO18B	ADCY2
NRAP	KAZN
POU6F2	DGKB
SHISA6	DDC
CA10	APBB2
TRDN	PLA2G4C
POU2F3	NOS1AP
ANO3	SLC7A13
UNC80	CADPS2
SLCO6A1	CADPS
STXBP5L	ABCA8
SNAP91	DPP10
MLPH	NKAIN1
EPB41L4B	KSR2
KIAA1549L	SYT14
MS4A13	REEP1
NXPE2	GALNT13
MGAT4C	CGNL1
ZBBX	SNX7
NPAS3	SNX31
ELAVL2	IQCJ
LIMCH1	LRRIQ1
LRRC49	MIPOL1
SERPINB11	RGL3
LRRC75B	LRRC31
CDC20B	ANKRD62

**Table A6:** Female-biased genes (FG) in CF found in StringDB associated with alternative splicing (StringDB term KW-0025).

Gene Name	Gene Name
AIRE	SLFN12L
TXLNG	ZNF711

**Table A7:** Male-biased (MG) alternatively spliced genes in CF patients.

Ensembl ID	Gene Name
ENSG00000122783	C7orf49
ENSG00000073578	SDHA
ENSG00000198034	RPS4X
ENSG00000031698	SARS
ENSG00000122783	C7orf49
ENSG00000175567	UCP2
ENSG00000115464	USP34
ENSG00000159140	SON
ENSG00000134905	CARS2
ENSG00000115760	BIRC6
ENSG00000168028	RPSA
ENSG00000236213	AC006369.1
ENSG00000162402	USP24
ENSG00000107290	SETX
ENSG00000228655	AC096558.1
ENSG00000141469	SLC14A1

**Table A8:** Female-biased (FG) alternatively spliced genes in CF patients.

Ensembl ID	Gene Name
ENSG00000058272	PPP1R12A
ENSG00000107099	DOCK8
ENSG00000055609	KMT2C
ENSG00000137642	SORL1
ENSG00000105851	PIK3CG
ENSG00000124942	AHNAK
ENSG00000197852	FAM212B
ENSG00000145416	MARCH1
ENSG00000183486	MX2
ENSG00000114331	ACAP2
ENSG00000160255	ITGB2
ENSG00000139083	ETV6
ENSG00000164631	ZNF12
ENSG00000118058	KMT2A
ENSG00000069667	RORA
ENSG00000101596	SMCHD1
ENSG00000197329	PELI1
ENSG00000106714	CNTNAP3
